# Effects of Oxygen Flow Rate on Metal-to-Insulator Transition Characteristics in NbO_x_-Based Selectors

**DOI:** 10.3390/ma15238575

**Published:** 2022-12-01

**Authors:** Osung Kwon, Hongmin Lee, Sungjun Kim

**Affiliations:** Division of Electronics and Electrical Engineering, Dongguk University, Seoul 04620, Republic of Korea

**Keywords:** selector, niobium oxide, oxygen flow rate, metal-to-insulator transition

## Abstract

In this work, NbOx-based selector devices were fabricated by sputtering deposition systems. Metal-to-insulator transition characteristics of the device samples were investigated depending on the oxygen flow rate (3.5, 4.5, and 5.5 sccm) and the deposition time. The device stack was scanned by transmission electron microscopy (TEM) and energy-dispersive X-ray spectroscopy (EDS). The yields, including MIT, nonlinear, and Ohmic, in working devices with different deposition conditions were also evaluated. Moreover, we observed the trend in yield values as a function of selectivity. In addition, the current–voltage (I–V) curves were characterized in terms of DC and pulse endurance. Finally, the switching speed and operating energies were obtained by applying a triangular pulse on the devices, and the recovery time and drift-free characteristics were obtained by the paired pulses.

## 1. Introduction

The ability to store information is important with the development of information technologies. With the fast development of electronic devices, flash memory, including NAND and NOR types, represents a nonvolatile memory due to its high-density properties. However, flash technology has several disadvantages, including high voltage operation and low switching speed [1]. Therefore, it is urgent to develop the next generation of nonvolatile memory for high-capacity storage, such as phase-change random-access memory (PRAM), magnetic random-access memory (MRAM), and resistive random-access memory (RRAM) [2,3,4,5,6,7]. Among them, RRAM can be a representative candidate for the next generation of nonvolatile memory because RRAM has several advantages [2,8,9,10,11,12,13,14]. It can offer low power consumption, fast switching speeds, high endurance, and high density with crossbar array integration.

RRAM with a crossbar array structure is considered a leading challenger for future computing paradigms, such as in-memory computing and neuromorphic computing. However, the crossbar array structure presents a sneak current problem that seriously affects misreading of information and memory operation [15]. Various studies have been conducted to solve this problem. First, the self-rectifying RRAM device is effective in solving the sneak current problem without the connection of an additional selector. Secondly, complementary resistive switching (CRS) was designed, composed of two RRAM stacks anti-serially connected [16,17]. The CRS devices can reduce the sneak current problem but cannot avoid read destruction. Thirdly, a nonlinear device was directly combined with the RRAM cell to effectively reduce the sneak current. Examples include the one-transistor one-resistor (1T1R) [15,18,19], one-diode one-resistor (1D1R) [15,20,21], and one-selector one-resistor (1S1R) [15,22] devices. In particular, the 1S1R structure is considered the best structure for RRAM with bipolar resistive switching, exhibiting excellent memory performance [15].

NbO_2_ [23,24] and ZnTe [25,26] are used in selector devices due to their stable threshold switching [27]. NbO_2_ has recently been studied in a wide range of applications, such as optical sensors and various electronic devices, due to its metal-to-insulator transition (MIT) characteristics [27]. Research has reported that it is possible to suppress leakage current in a crossbar array structure by combining an RRAM cell with a NbO_x_-based selector device with MIT characteristics [27]. TiN was used as the top and bottom electrodes to induce more oxygen vacancies, due to its high oxygen reservoir content [28,29].

In this study, we have fabricated NbO_x_-based selector devices using reactive sputtering. A total of nine devices were investigated by investigating different sputtering times of 7, 10, and 13 min and differing the oxygen flow rate from 3.5 to 4.5 to5.5 sccm for statistical analysis. We obtained the yield for the devices made with different thicknesses and oxygen flow rates. The TiN/NbOx/TiN devices were verified using energy-dispersive X-ray spectroscopy (EDS) and transmission electron microscopy (TEM). In addition, the current–voltage (I–V) curves were characterized by measuring DC and AC endurance. The switching speed was measured in detail using a triangular pulse to check the operating energy, and the recovery time and drift-free characteristics were investigated.

## 2. Experiments

TiN/NbOx/TiN selector devices were fabricated as follows: First, a 100 nm thick TiN bottom electrode (BE) was deposited on a SiO_2_/Si wafer by reactive sputtering. Ar (19 sccm) and N_2_ (1 sccm) were used at a pressure of 3 mTorr and RF power of 280 W for the TiN film with a Ti target and pure N_2_ gas. Then, a NbOx layer was deposited by RF reactive sputtering with an Nb target at room temperature and a pressure of 5 mTorr. NbOx layers with various compositions were obtained by controlling the oxygen flow. The flow rate of Ar/O_2_ was 20/3.5 sccm (14.89%), 20/4.5 sccm (18.37%), and 20/5.5 sccm (21.57%), respectively. Finally, a 100 nm thick TiN top electrode (TE) was defined by lithography and a lift-off process by reactive sputtering in the same way as the bottom electrode. A Keithley 4200-SCS semiconductor parameter analyzer (SPA) and a 4225-PMU pulse measurement unit in the probe station were used to measure electrical characteristics using DC sweep mode and transient characteristics. A bias was applied to the TiN (top electrode) and the TiN (bottom electrode) was grounded.

## 3. Results and Discussion

**Figure 1:**
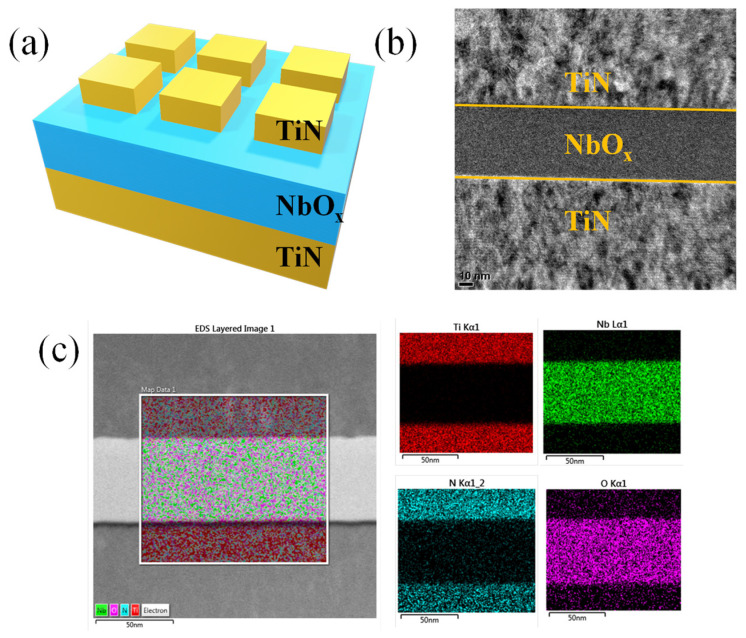
Stack of the TiN/NbOx/TiN device: (**a**) schematic and (**b**) TEM image. (**c**) EDS color mappings for the elements Ti, Nb, N, and O.

Figure 1a shows a schematic of the fabricated TiN/NbOx/TiN selector device. A cross-sectional TEM image of the TiN/NbOx/TiN device is presented in Figure 1b, and the elementary distribution is revealed by EDS in Figure 1c. Note that the EDS mapping confirmed the composition of the device stack.

**Figure 2:**
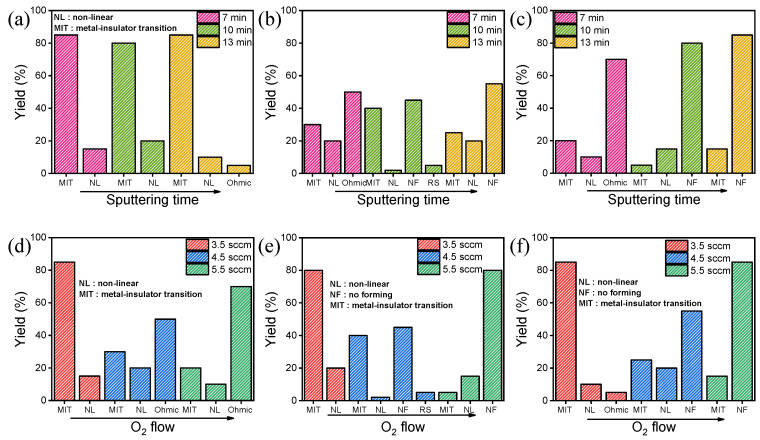
MIT characteristic yield of TiN/NbOx/TiN devices with different oxygen flow rates: (**a**) 3.5 sccm, (**b**) 4.5 sccm, and (**c**) 5.5 sccm. MIT characteristic yield of TiN/NbOx/TiN devices with different sputtering times: (**d**) 7 min, (**e**) 10 min, and (**f**) 13 min.

Figure 2a–c shows the yield with the different sputtering times with an oxygen flow rate of 3.5 sccm, 4.5 sccm, and 5.5 sccm, respectively. Twenty cells were measured to collect the yield data for each sample. Figure 2a shows the yield of the TiN/NbOx/TiN device produced at an oxygen flow rate of 3.5 sccm. The yields are approximately 80% for the MIT characteristic after 7, 10, and 13 min sputtering time. Figure 2b shows the yield of the devices made at an oxygen flow rate of 4.5 sccm in which the MIT characteristic yields are between 25% and 40%. Figure 2c shows the devices produced at an oxygen flow rate of 5.5 sccm, in which MIT characteristic yields are 20% or less. In Figure 2b,c, the devices deposited for 7 min show mostly Ohmic characteristics, but the devices deposited for 10 or 13 min show an increasing proportion of cells that are not included in the forming process. Based on the above experimental results, the MIT properties do not change significantly even if the thickness deposited varies by varying the sputtering time at the same flow rate. The NbO_x_-based selector device is thickness-independent. Figure 2d–f shows the yield as a function of the oxygen flow rate at the deposition time of 7, 10, and 13 min, respectively. In Figure 2d–f, there was a change in the MIT properties when the oxygen flow was different and when the deposition time was unchanged. However, they may have different thicknesses due to different sputtering deposition conditions, so further research is needed.

**Figure 3:**
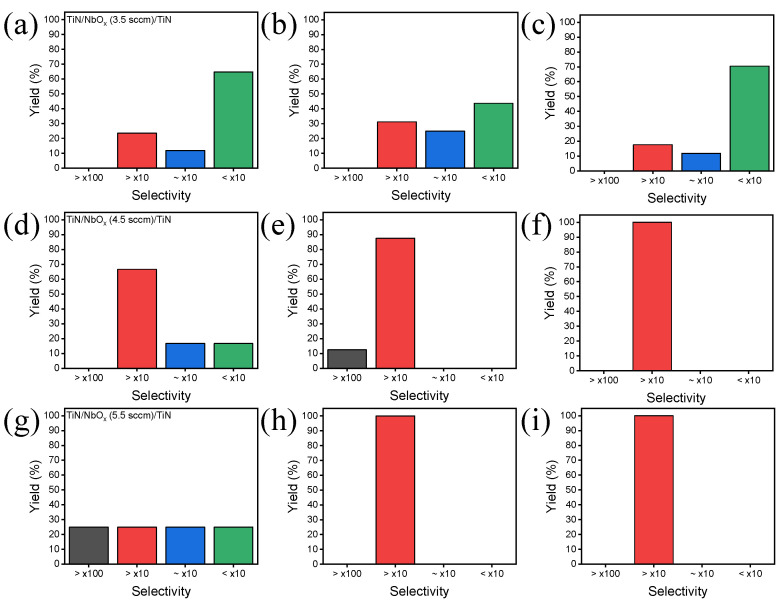
Selectivity of TiN/NbOx/TiN devices: (**a**) 3.5 sccm, 7 min, (**b**) 3.5 sccm, 10 min, (**c**) 3.5 sccm, 13 min, (**d**) 4.5 sccm, 7 min, (**e**) 4.5 sccm, 10 min, (**f**) 4.5 sccm, 13 min, (**g**) 5.5 sccm, 7 min, (**h**) 5.5 sccm, 10 min, and (**i**) 5.5 sccm, 13 min (oxygen flow, sputtering time).

Figure 3a–j shows the selectivity of cells that exhibited MIT characteristics in nine devices at different sputtering times and oxygen flow rates. Figure 3a–c shows the yield of the selectivity of the device with an oxygen flow rate of 3.5 sccm at different sputtering times of 7, 10, and 13 min, respectively. All three devices show the most cells with a selectivity of <×10 (Figures S1–S3). Figure 3d–f shows the selectivity of the device at an oxygen flow rate of 4.5 sccm, and it was confirmed that the selectivity was greater than that of the oxygen flow rate of 3.5 sccm (Figures S4–S6). Furthermore, it was confirmed that devices made at an oxygen flow rate of 5.5 sccm also had greater selectivity than devices made at 3.5 sccm (Figures S7–S9) in Figure 3g–i. Note that for NbOx deposited with an increasing the oxygen flow rate, the MIT characteristic yield decreased but the selectivity increased.

**Figure 4:**
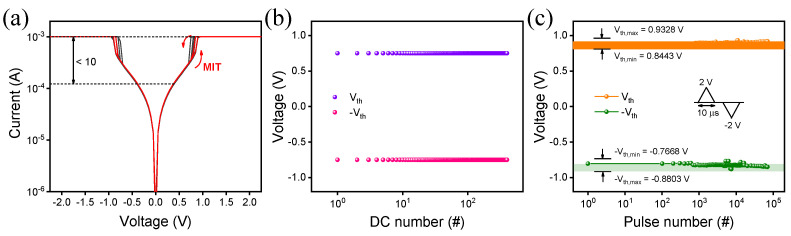
(**a**) I−V curve of MIT characteristics. The endurance test: (**b**) DC sweep and (**c**) pulse mode.

Figure 4a shows I−V curves with MIT characteristics in a TiN/NbOx/TiN device operating as a selector to suppress leakage current in a crossbar array structure. A dual sweeping from 0 to 3 V in the positive region is needed for the device to work. The current rapidly increases from about 0.8 V with a compliance current (CC) of 1 mA. Here, the turn-on voltage is called the threshold voltage (V_th_). Subsequently, the current rapidly decreases again at about 0.6 V. Here, the hold voltage (V_h_) is defined as the turn-off voltage. Similarly, when a voltage sweep is performed in the negative region, the current increases rapidly from around −0.8 V with a CC of 1 mA, and the current decreases rapidly again at around −0.6 V. The turn-on and turn-off voltages are defined as −V_th_ and −V_h_, respectively. Figure 4b shows 300 cycles of DC endurance characteristics for the device that sputtered at 3.5 sccm for 7 min. Importantly, the device had excellent uniformity and stability during the 300 cycles of DC measurements. Figure 4c shows pulse endurance characteristics of the device sputtered at 3.5 sccm for 7 min. For 70,000 cycles, a positive V_th_ has a low variation range between 0.93 V (V_th_max_) and 0.84 V (V_th,min_), while a negative V_th_ is located from −0.77 V (−V_th,max_) and −0.88 V (−V_th,min_). NbOx-based devices have good switching characteristics in terms of endurance and tight switching variation because of Joule heating-induced filamentary metal-to-insulator transitions.

**Figure 5:**
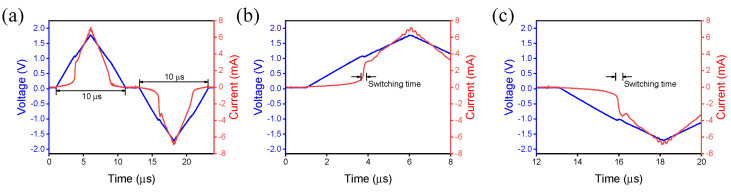
(**a**) The triangular pulse with 10 μm of pulse width, (**b**) switching time at positive bias, (**c**) switching time at negative bias.

We applied triangular pulses on the device to measure the intrinsic insulating and metallic states of the NbO_x_ selector device [30]. If the MIT in NbO_x_ is initiated by an electrical field, a sufficient current density is equated in the filament to complete insulating-monoclinic phases to a metallic rutile phase assisted by Joule heating [30,31]. Figure 5a shows the transient characteristics by set and reset pulses. A pulse amplitude of 2 V and a pulse width of 10 µs were used for switching. The threshold switching speed is measured in the positive region in Figure 5b. The current increased rapidly when the voltage of the triangular pulse was around 1 V, and the rising time was calculated to obtain the switching speed. Similarly, the threshold switching speed was obtained in the negative bias in the same way, as shown in Figure 5c. The switching energy is obtained by Equation (1) as

(1) $$Switching\;Energy=I_{CC}\times U\times t_{switching}$$

where $I_{CC}$ is the compliance current, U is the pulse amplitude, and $t_{switching}$ is the switching time [32,33]. As a result of obtaining the switching energy of a TiN/NbOx/TiN device using the threshold switching speed, it was confirmed that this device has a low switching energy of 70 to 80 pJ (Figure S10).

**Figure 6:**
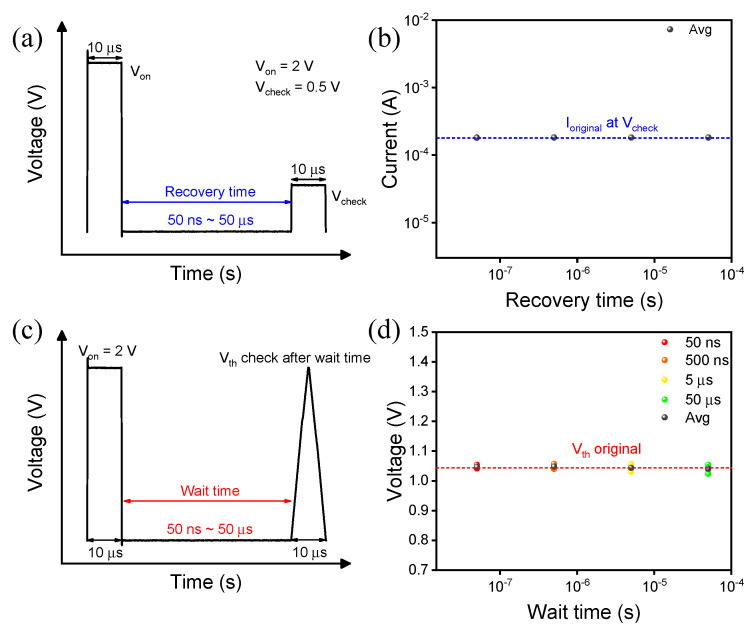
Recovery characteristics of the TiN/NbO_x_/TiN device: (**a**) pulse and (**b**) recovery time. Drift characteristics of the TiN/NbO_x_/TiN device: (**c**) pulse and (**d**) drift-free.

Next, we obtained the recovery time of the device from the metal phase to the insulating phase [34]. The device at 3.5 sccm and sputtering time of 7 min can be recovered in below 20 ns. To record the switching speed, the device was biased with a 2 V pulse, the recovery time range was set at 50 ns to 50 μs (Figures S11−S13), and the current was recorded at V_check_ (0.5 V pulse) in Figure 6b. A steady current was observed below the recovery time of 50 ns. To investigate the drift-free operation, we applied a 2 V pulse with different wait times ranging from 50 μs to 50 ns (Figures S15−S18). Figure 6c shows the drift-free operation without changing V_th_ due to the difference in pulse time intervals [34]. Figure 6d monitors the voltage as a function of wait time. The results show that the switching mechanism is primarily related to a MIT [27,34].

## 4. Conclusions

In this article, TiN/NbOx/TiN devices were prepared by sputtering for selector application in a crossbar array. An MIT was confirmed with the high slope of I−V in the devices, ensuring selector device characteristics. The oxygen flow rate (3.5, 4.5, and 5.5 sccm) and the deposition time were controlled to obtain the optimal MIT characteristics. The device stack and its chemical deposition were verified by TEM and EDS analysis. The yields, including MIT, nonlinear, and Ohmic, were investigated. Through the measured data, it was confirmed that the MIT characteristics were well exhibited in the device fabricated with an Ar/O_2_ ratio of 20/3.5 sccm. Furthermore, the yield values as a function of selectivity were investigated. As the oxygen flow rate increases, the selectivity increases, but the yield of MIT characteristics decreases. In addition, typical current−voltage (I−V) curves were presented, and good DC and pulse endurance were found. Moreover, the switching speed and operating energies were calculated by triangular pulses. Finally, the recovery time and wait time were calculated to understand the MIT characteristics.

## Data Availability

Not applicable.

## Supplementary Materials

The following supporting information can be downloaded at: https://www.mdpi.com/article/10.3390/ma15238575/s1.
