# Supplementary material for: Effects of Oxygen Flow Rate on Metal-to-Insulator Transition Characteristics in NbOx-Based Selectors
Source: Materials (Basel). 2022 Dec 1;15(23):8575. doi: 10.3390/ma15238575 (PMC9739534; doi:10.3390/ma15238575)
Supplement: Supplementary file 1 [file materials-15-08575-s001.zip › materials-2020733-supplementary.pdf]

Supporting information:

Effect of Oxygen Flow Rate on Metal-to-Insulator Transition Characteristics in NbO_x-Based Selectors

Osung Kwon †, Hongmin Lee †, Sungjun Kim *

Division of Electronics and Electrical Engineering, Dongguk University, Seoul 04620, Korea

† Correspondence: sungjun@dongguk.edu

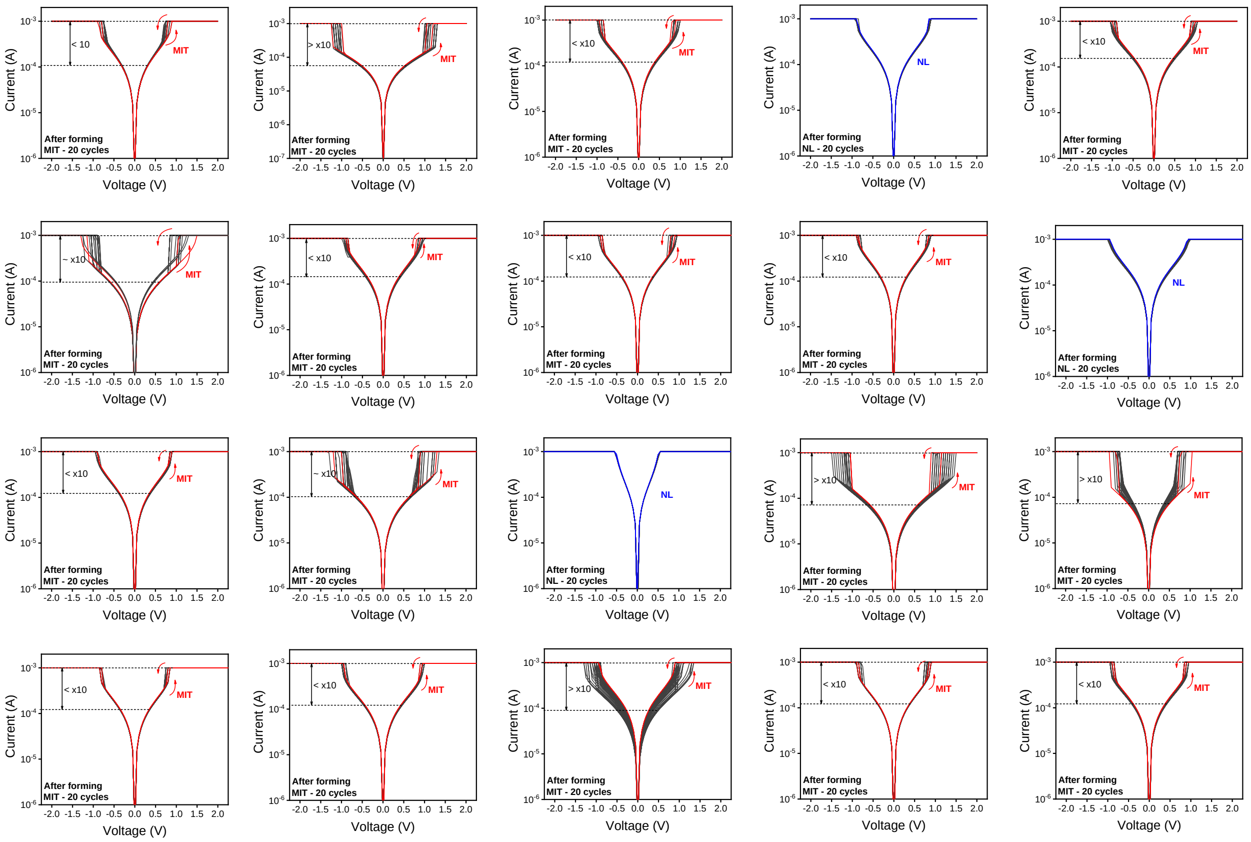

Figure S1. I-V curve measuring MIT characteristics in 20 cells of a device fabricated by depositing 3.5 sccm of oxygen flow for 7 min.

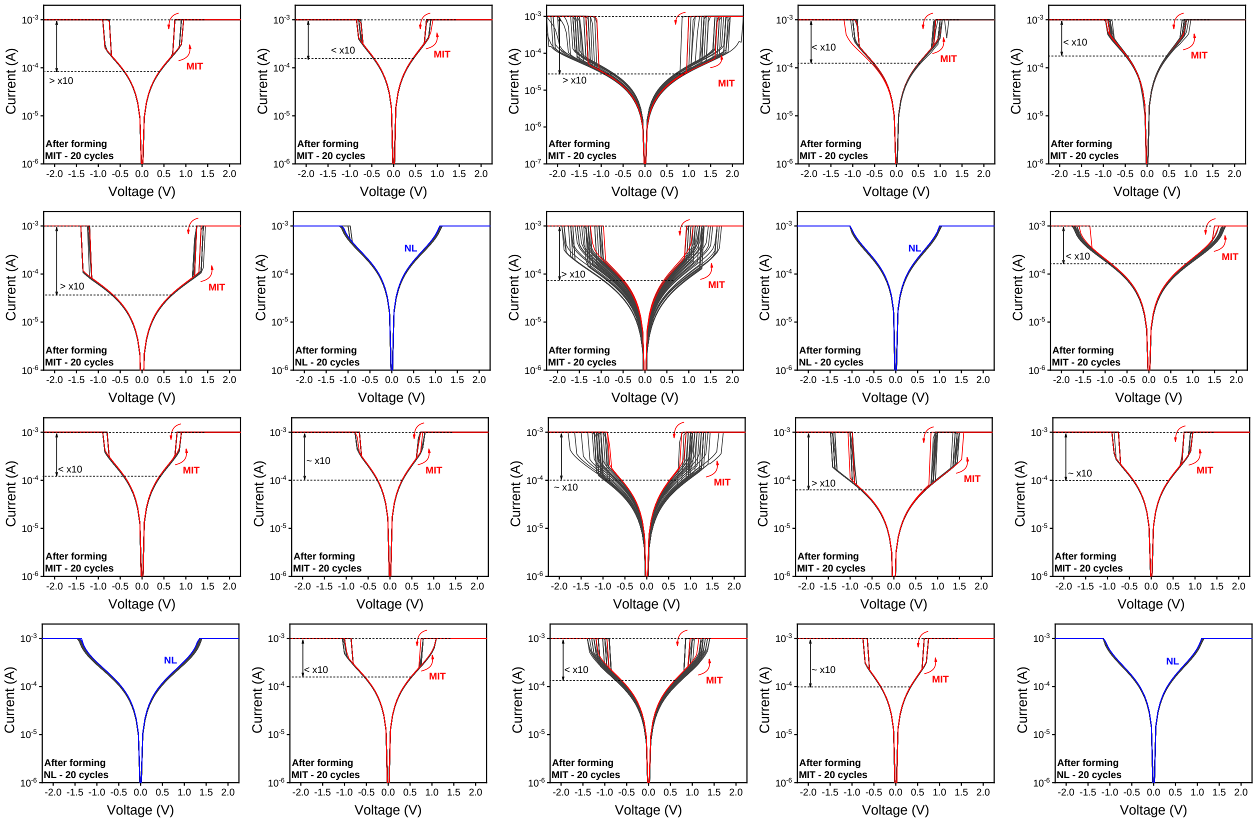

Figure S2. I-V curve measuring MIT characteristics in 20 cells of a device fabricated by depositing 3.5 sccm of oxygen flow for 10 min.

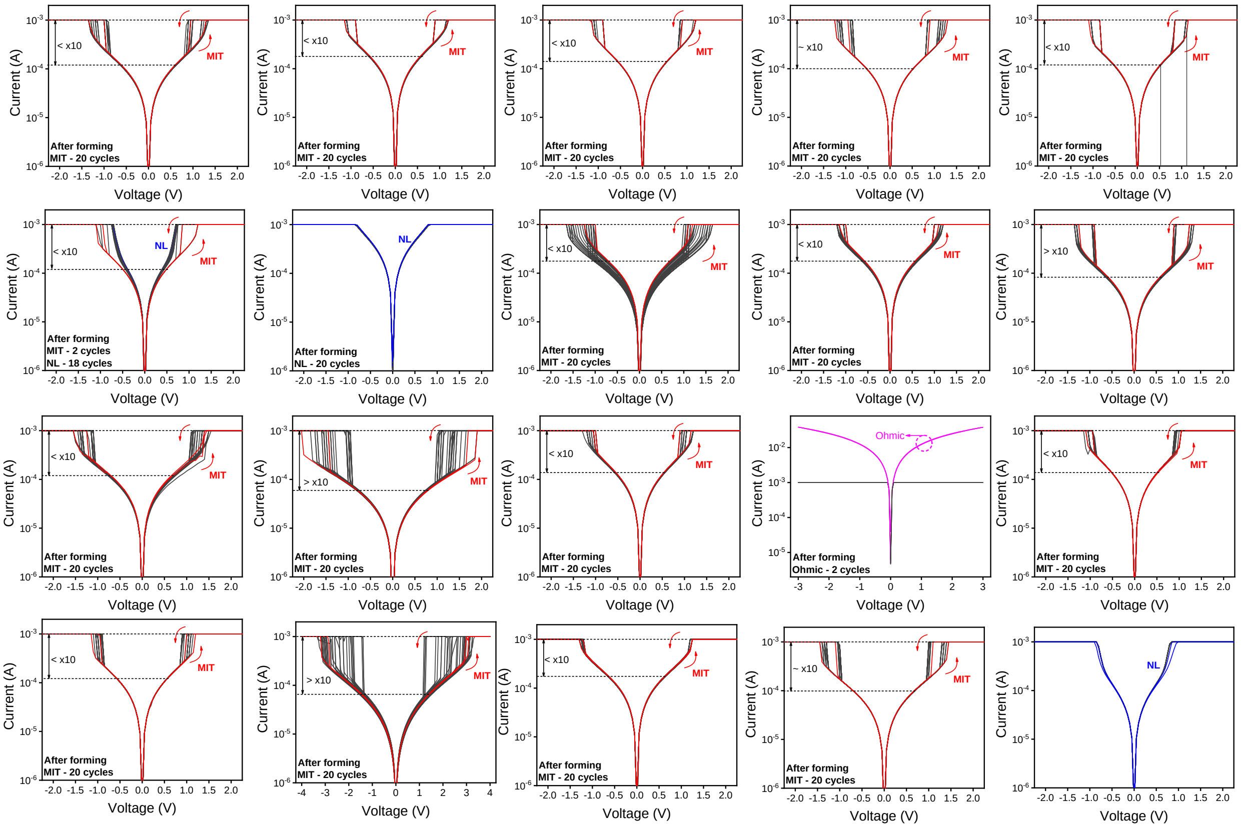

Figure S3. I-V curve measuring MIT characteristics in 20 cells of a device fabricated by depositing 3.5 sccm of oxygen flow for 13 min.

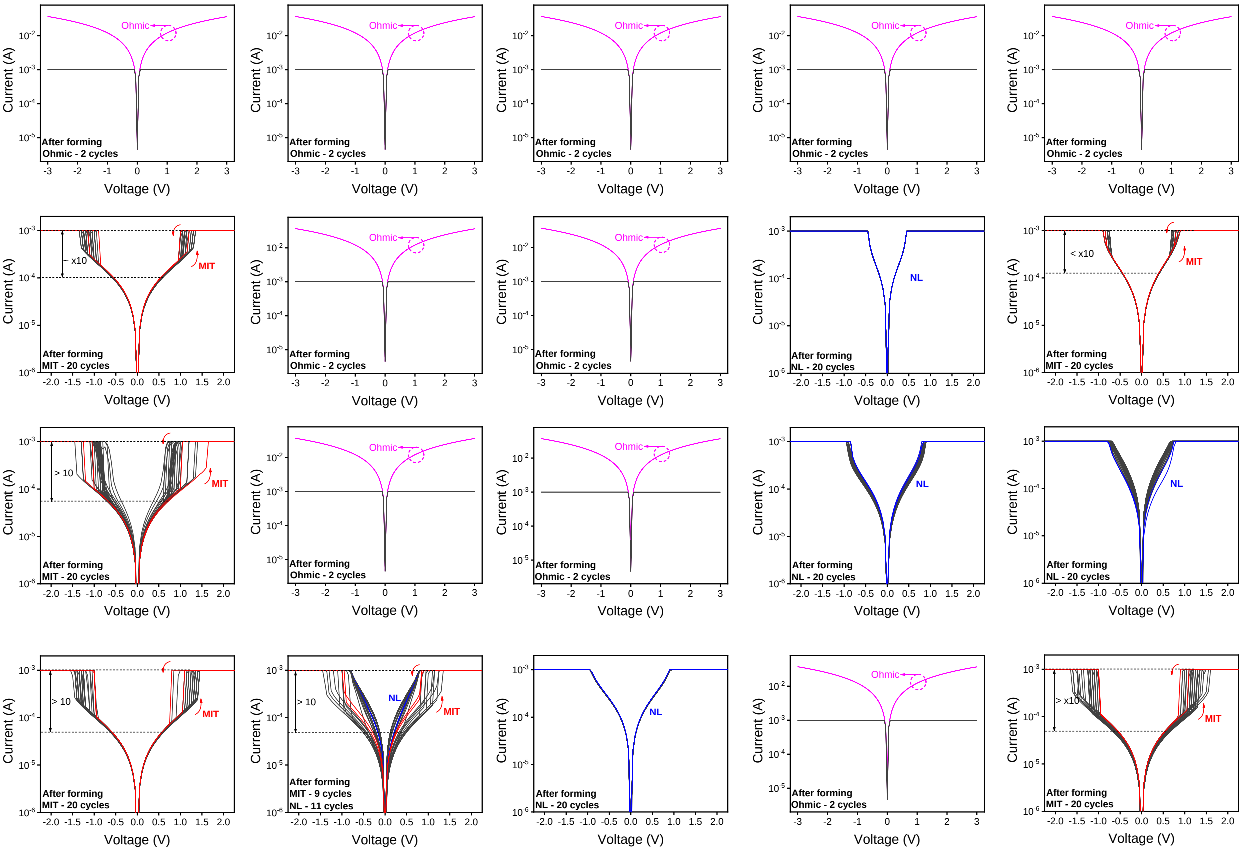

Figure S4. I-V curve measuring MIT characteristics in 20 cells of a device fabricated by depositing 4.5 sccm of oxygen flow for 7 min.

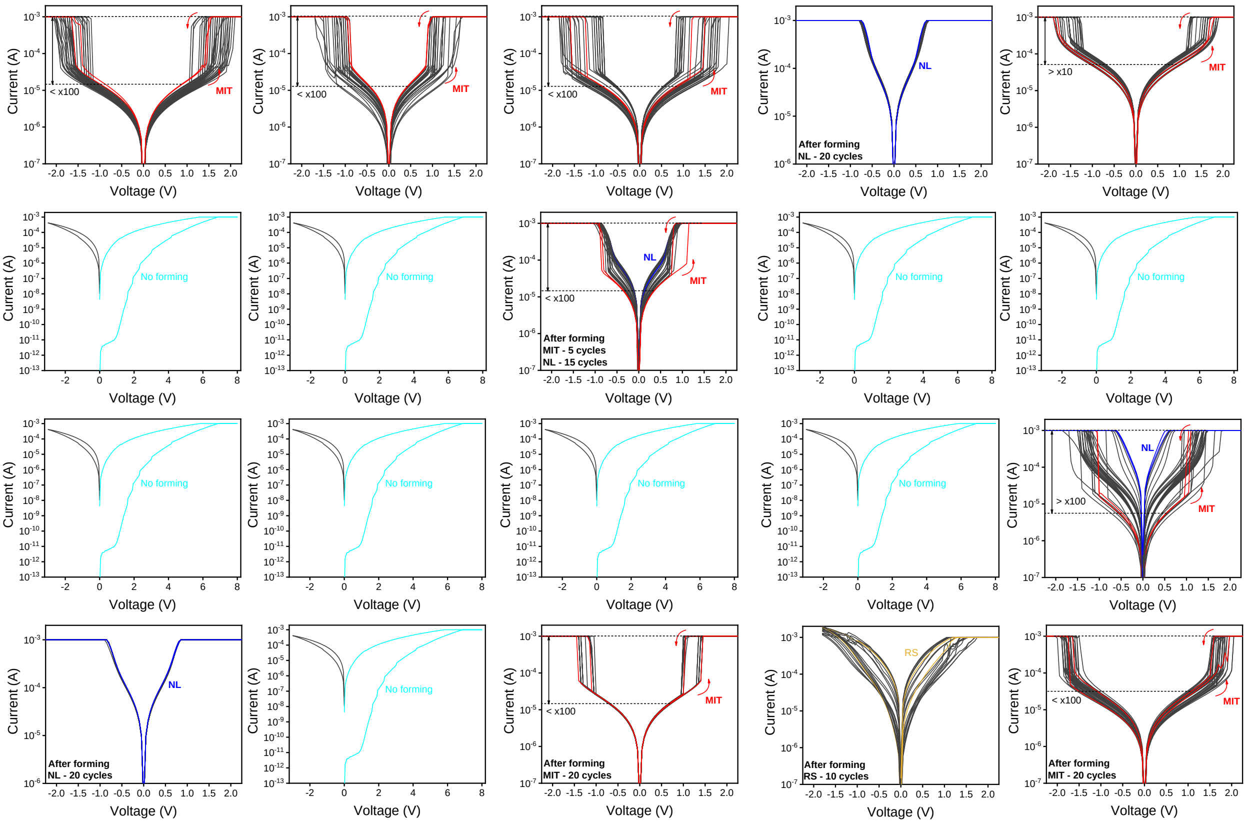

Figure S5. I-V curve measuring MIT characteristics in 20 cells of a device fabricated by depositing 4.5 sccm of oxygen flow for 10 min.

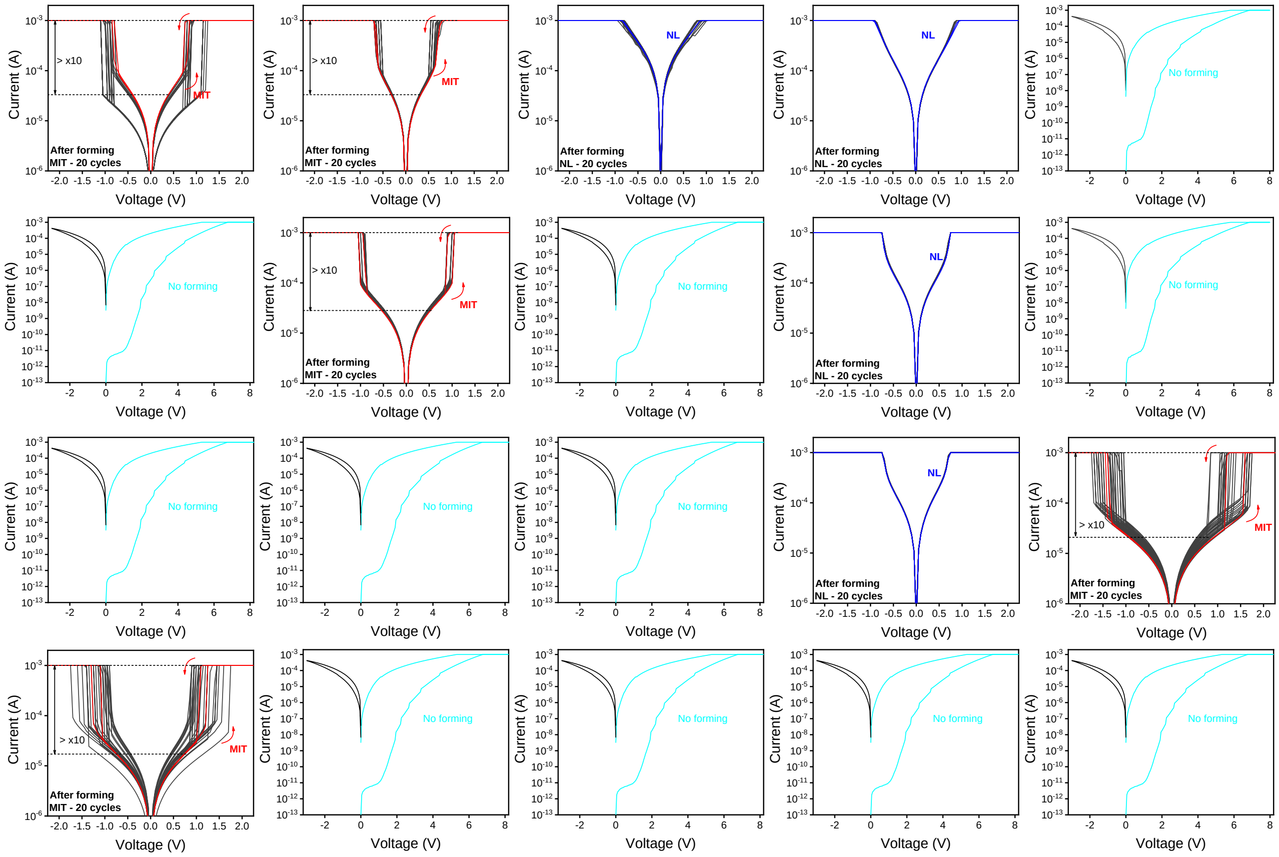

Figure S6. I-V curve measuring MIT characteristics in 20 cells of a device fabricated by depositing 4.5 sccm of oxygen flow for 13 min.

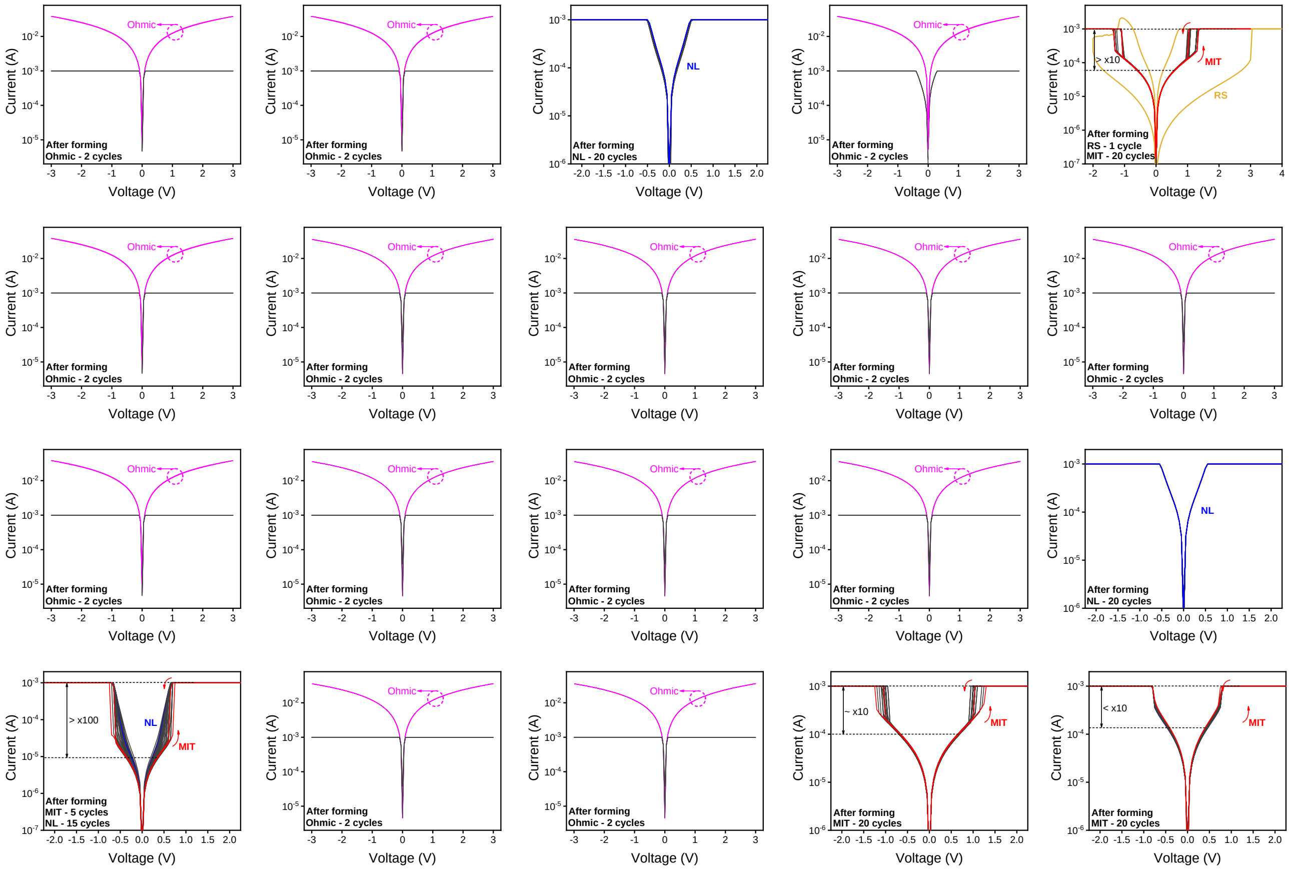

Figure S7. I-V curve measuring MIT characteristics in 20 cells of a device fabricated by depositing 5.5 sccm of oxygen flow for 7 min.

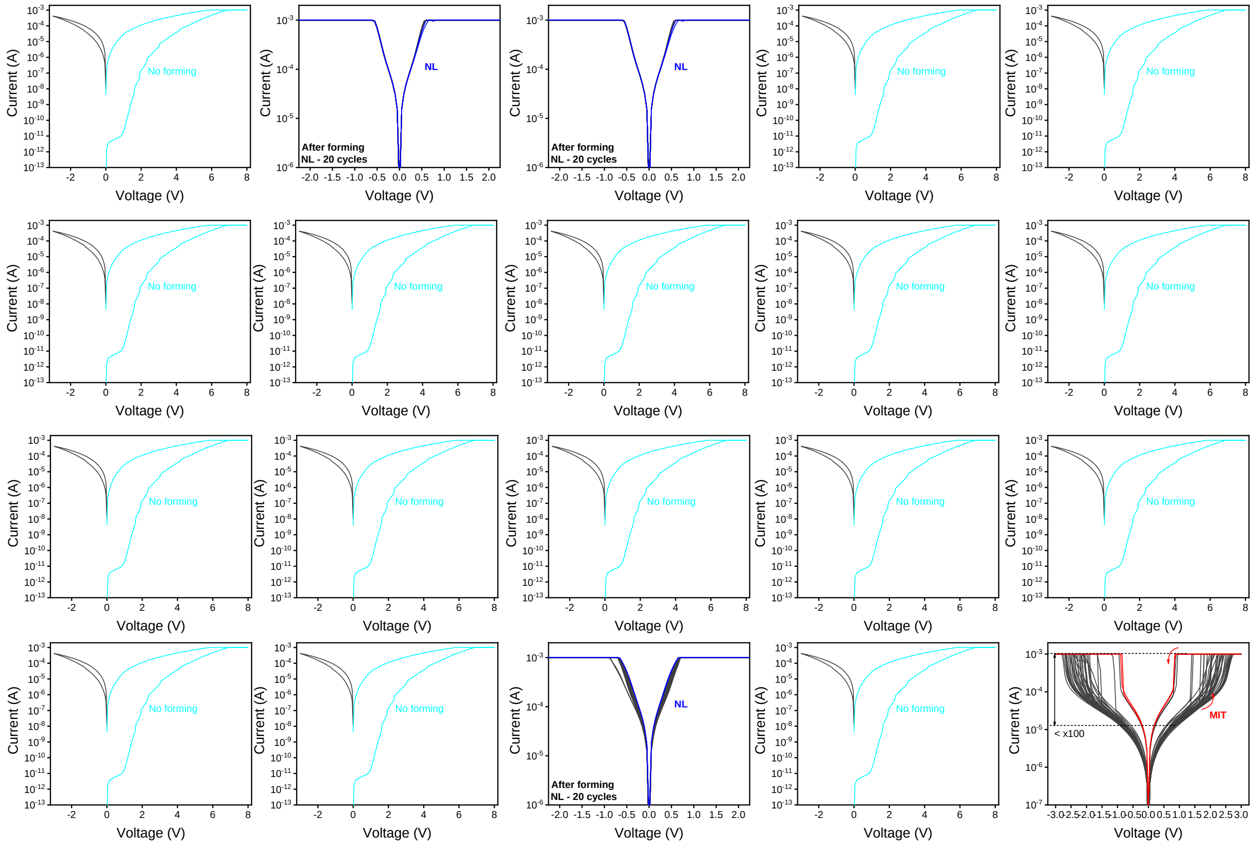

Figure S8. I-V curve measuring MIT characteristics in 20 cells of a device fabricated by depositing 5.5 sccm of oxygen flow for 10 min.

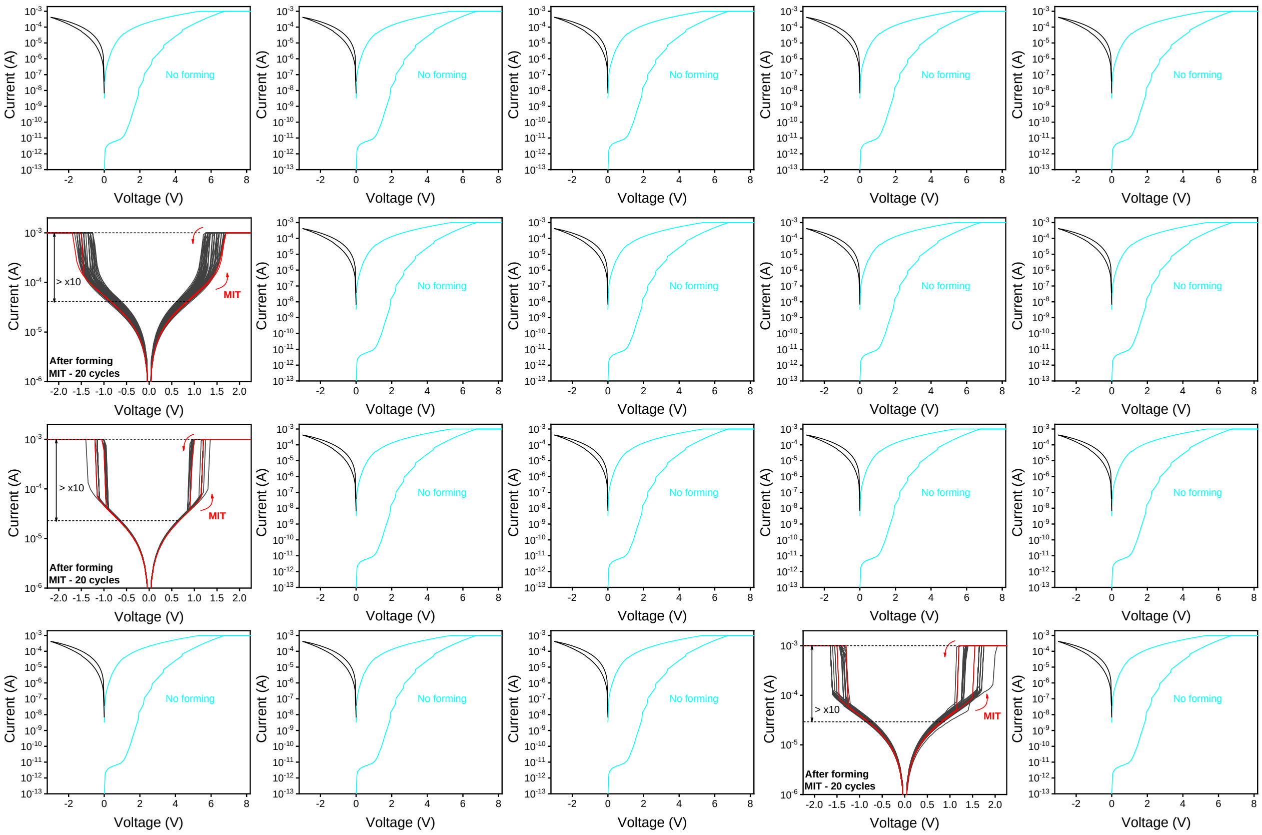

Figure S9. I-V curve measuring MIT characteristics in 20 cells of a device fabricated by depositing 5.5 sccm of oxygen flow for 13 min.

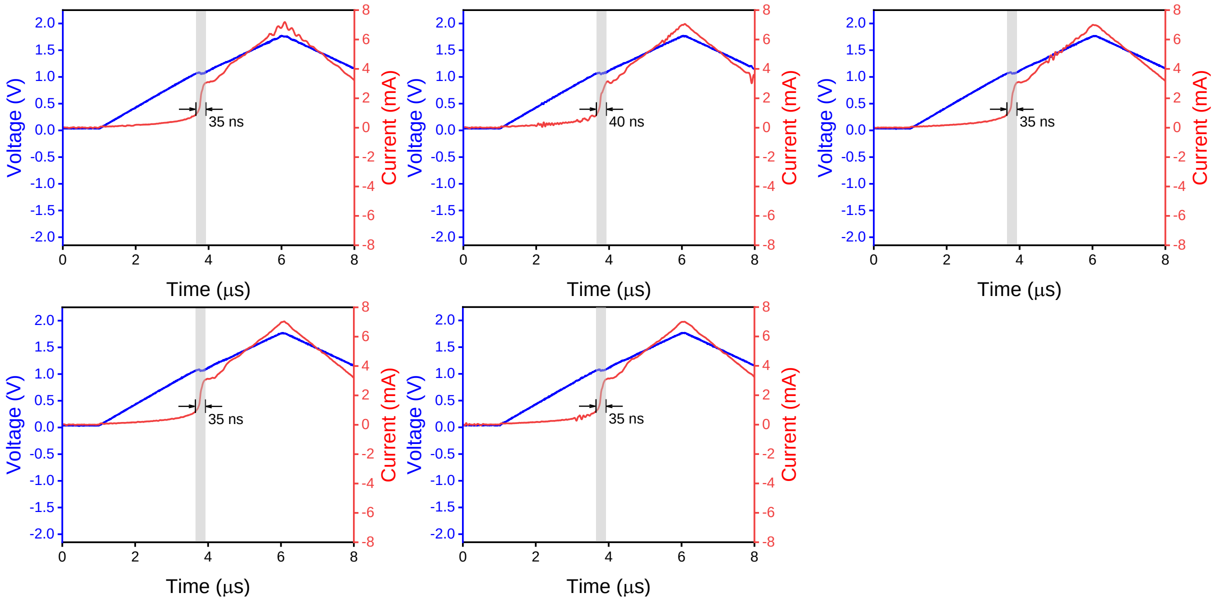

Figure S10. Switching time at 5 cells of a device deposited at oxygen flow rate of 3.5 sccm for 7 min.

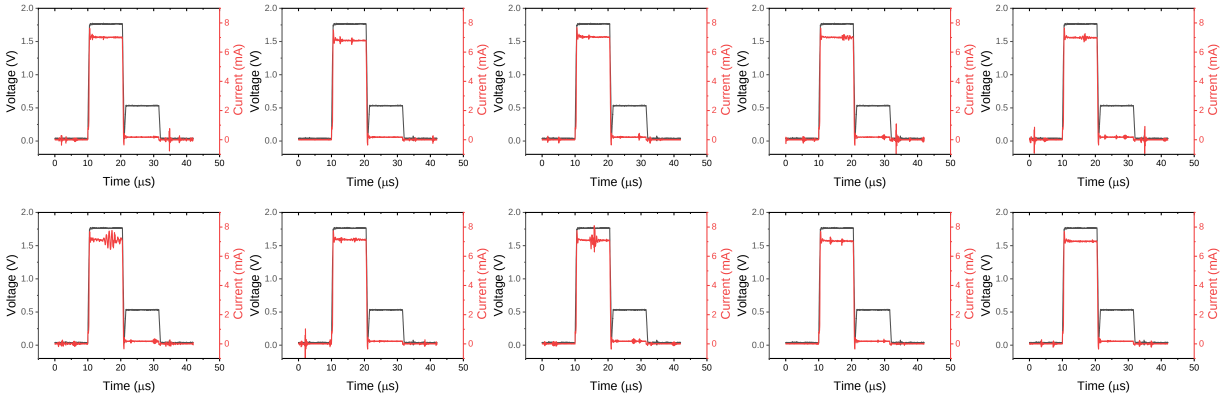

Figure S11. Recovery characteristics (50 ns) in 20 cells of a device fabricated by depositing 3.5 sccm of oxygen flow for 7 min.

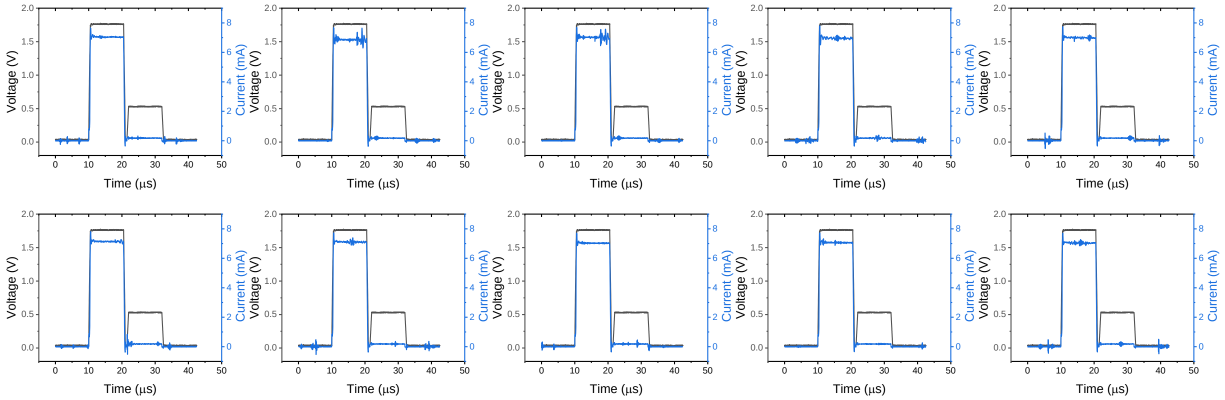

Figure S12. Recovery characteristics (500 ns) in 20 cells of a device fabricated by depositing 3.5 sccm of oxygen flow for 7 min.

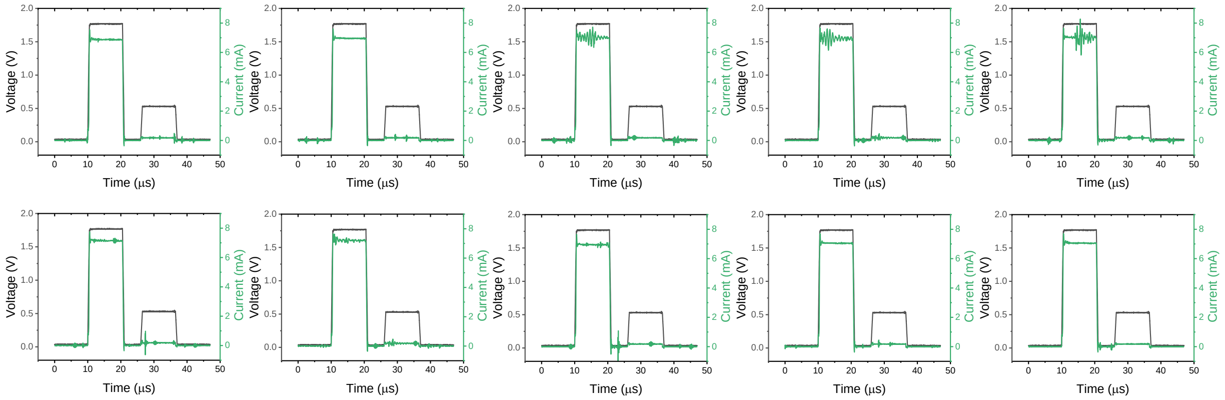

Figure S13. Recovery characteristics (5 µs) in 20 cells of a device fabricated by depositing 3.5 sccm of oxygen flow for 7 min.

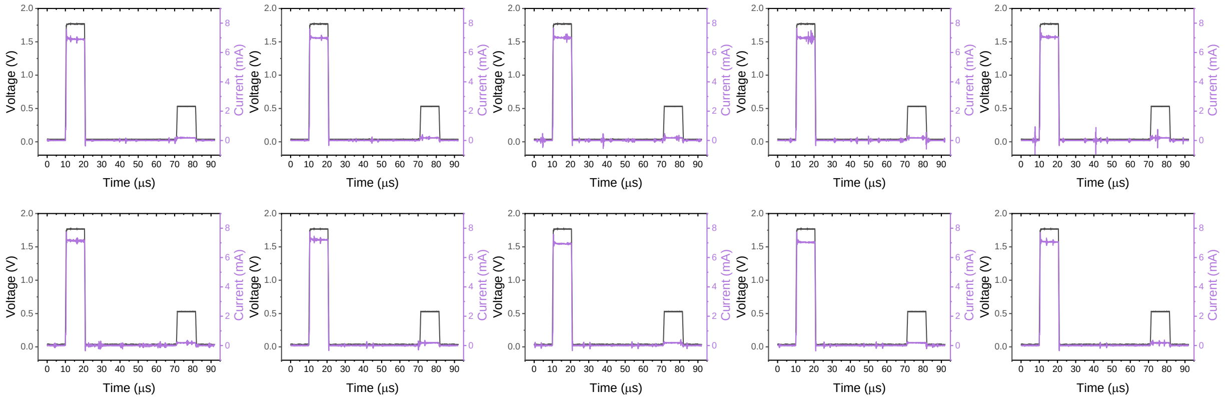

Figure S14. Recovery characteristics (50 µs) in 20 cells of a device fabricated by depositing 3.5 sccm of oxygen flow for 7 min.

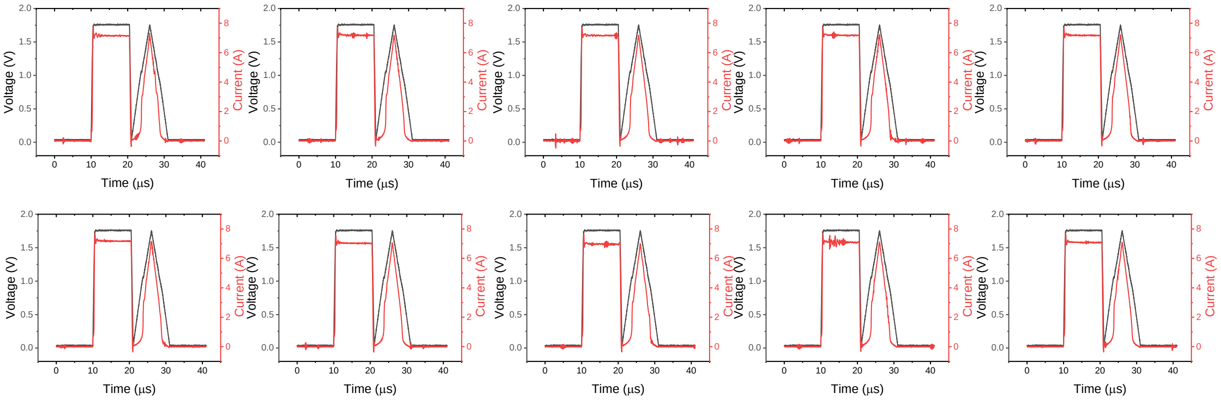

Figure S15. Drift characteristics (50 ns) in 20 cells of a device fabricated by depositing 3.5 sccm of oxygen flow for 7 min.

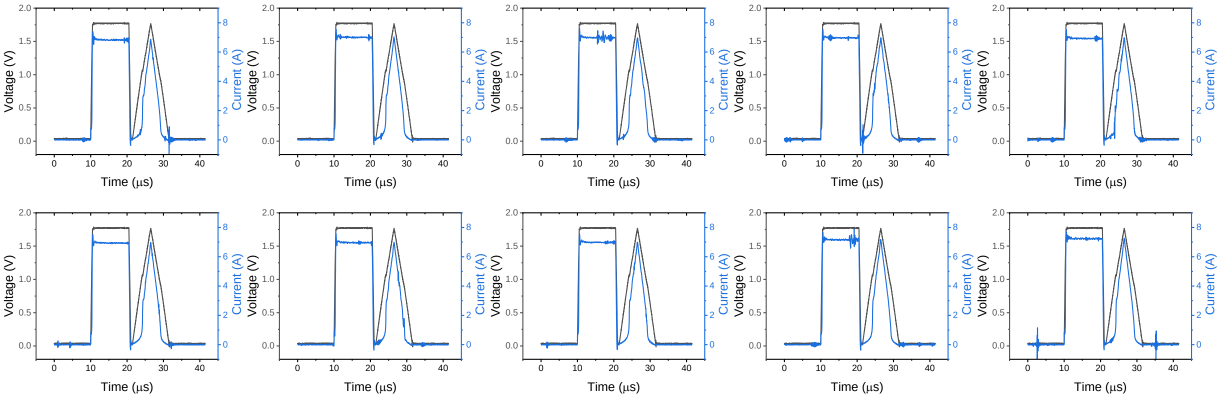

Figure S16. Drift characteristics (500 ns) in 20 cells of a device fabricated by depositing 3.5 sccm of oxygen flow for 7 min.

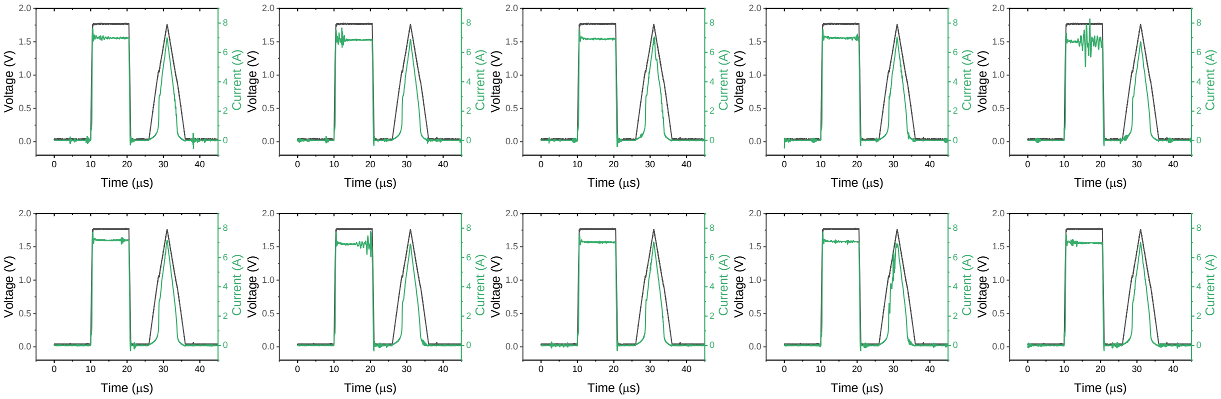

Figure S17. Drift characteristics (5 μs) in 20 cells of a device fabricated by depositing 3.5 sccm of oxygen flow for 7 min.

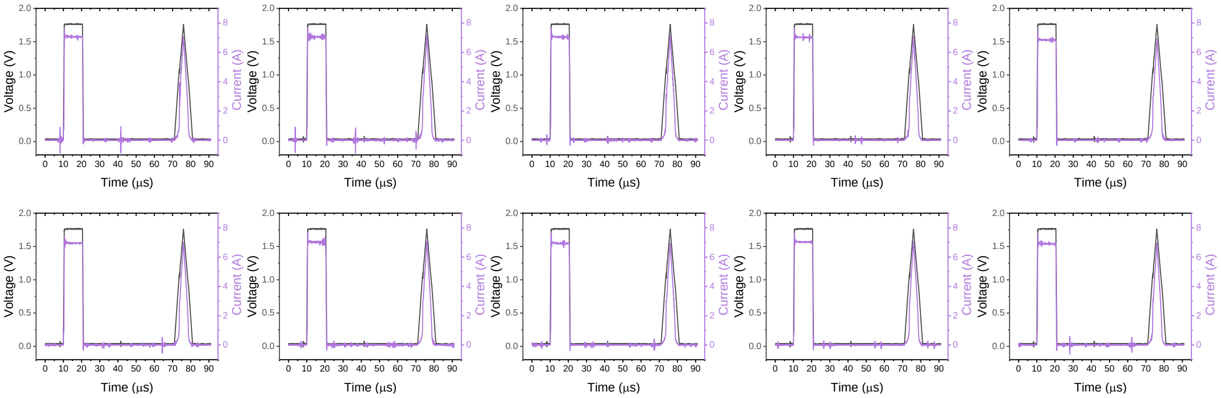

Figure S18. Drift characteristics (50 μs) in 20 cells of a device fabricated by depositing 3.5 sccm of oxygen flow for 7 min.
